# Social distancing using IoT approach

**DOI:** 10.1186/s43067-021-00040-z

**Published:** 2021-11-10

**Authors:** Mayuri Diwakar Kulkarni, Khalid Alfatmi, Nikhil Sunil Deshmukh

**Affiliations:** 1 SVKM’s Institute of Technology, Dhule, India; 2 SSVPs BSD College Of Engineering, Dhule, India

**Keywords:** COVID-19, WHO, Social distancing, IoT, PIR, Etc

## Abstract

In the coronavirus outbreak pandemic by COVID-19, the World Health Organization (WHO) has been issuing several guidelines through all government agencies. In line with those guidelines, social distancing in the population has been a major prevention practice, compelled by all government agencies worldwide. Despite strong recommendations to maintain at least one-and-a-half-meter distance between the persons, the guideline is not scrupulously followed. To overcome this situation, an IoT-based technical solution is proposed through this paper. PIR sensor is used for the detection of a target in the vicinity (1.5 m). Upon violation of social distancing norms, the system will trigger an audio alarm after the detection of the target object. The research paper model is prepared by considering the needs of the people. Many researchers are focusing on tracking affected persons, but few are focusing on the social distancing preventive. The suggested portable device will always notify the person who is violating the norm of 1.5 m. The proposed device will minimize the possibility of transmission and reduce the infection rate of COVID-19. The device uses a PIR sensor depending upon the applicability area of the human being.

## Introduction

COVID-19 (Coronavirus disease 2019) is an infectious disease caused by SARS-CoV-2 (severe acute respiratory syndrome coronavirus 2). It was identified in December 2019 in China. It was declared a pandemic by WHO. COVID-19’s doubling rate is on average 7.4 days [1]. The spread of COVID-19 is due to the transmission of coronavirus [2]. Coronavirus enters the human body through openings like the mouth, nose, and eyes. The droplets exerted through sneezing, coughing, and sometimes talking can spread the coronaviruses from person to person [2, 3].

To reduce the rate of COVID-19 transmissions, many government medical bodies and WHO have suggested some preventive measures through the guidelines. One of the important guidelines suggested by WHO to reduce transmission is social distancing. Social distancing indicates maintaining the distance between two persons. It is strongly recommended by WHO that a minimum distance of 1.5 m must be maintained to reduce COVID-19 transmission [4–7].

The proposed methodology in this paper is used to maintain a social distance of 1.5 m in the vicinity. Various approaches were suggested by the researchers. Researchers used the CCTV cameras [8] either at a public place or through the surveillance system through the Drone to check the crowd status in public places. Based on the crowd, governing authorities decide for maintaining the social distancing and preventive measures to reduce the spread of COVID-19. But these approaches are applicable in a mass population. At the same time, it is a responsibility of an individual to follow the interim guidelines issued by the WHO to maintain social distancing of 2 m. For the same, there is no such technical mechanism. Due to this, there is a need for such a device that will find the presence of human beings in the vicinity and alerts individually about the same so that he/she will maintain a social distancing.

Based on this approach, in the proposed work the wearable device is suggested. This device will help each individual to maintain at least social distancing. Due to this, the spread of the COVID-19 virus will minimize. In the suggested proposed device, PIR sensor interfaced with Arduino which will detect the human presence. If human presence will be there, then it will notify the individual through the audio message. Hence, the individual will be alerted and will maintain the social distancing at the public place also. By wearing this device, any person will come to know the presence of a human being nearby him/her. Due to that, it will be helpful to keep social distancing to avoid the spread of the COVID-19 virus.

By using this device, the user will be alerted in case of violation of the interim guideline issued by the WHO to maintain the social distancing. This will minimize the spread of the virus in the community or in the people who are infected or were in contact with infected persons or COVID-19-affected persons.

The paper comprises a literature survey related to COVID-19, guidelines to avoid the spread of COVID-19 followed by the scientific and technical approach used to avoid the spread of COVID-19 in a society in the motivation section. The method specifies the economically feasible solution provided by the usage of the PIR sensor to avoid the spread of the virus. The proposed algorithm specifies the working principle of the device designed by using a PIR sensor to indicate the human presence in a vicinity. The discussion section describes the different used techniques such as the Aarogya Setu app to alert the society about the infected person through the color schemes only. So what are the drawbacks associated with it? In the result section, the simulated results are shown followed by the conclusion.

## Literature review

WHO has released guidelines for the prevention of coronavirus transmission. These guidelines are circulated to the health ministers and health system administrators along other decision-makers of different countries. Also, the WHO guideline document ensures that COVID-19 patients can access life-saving treatment without compromising public health objectives. WHO released interim guidelines which are needed to be followed by the public to reduce the spread of COVID-19. These interim guidelines are for the preparedness and response plan for all the stages in COVID-19. According to guidelines, there must be 1.5 m distance among persons [4]. The main objective of WHO is to prevent an outbreak, delay spread, slow, and stop transmission [5].

To achieve this objective of WHO has strongly suggested social distancing.

According to the Center for Disease Control and Prevention, the Social distancing term is very extensive. The extensiveness of social distancing is in terms of closing of schools and workplaces, isolation, restricting the movement of people, and the cancellation of mass gathering [9].

According to Johns Hopkins Medicine, social distancing is also known as physical distancing. This is one of the guidelines laid by the WHO to prevent the spread of COVID-19 [4].

**Fig. 1:**
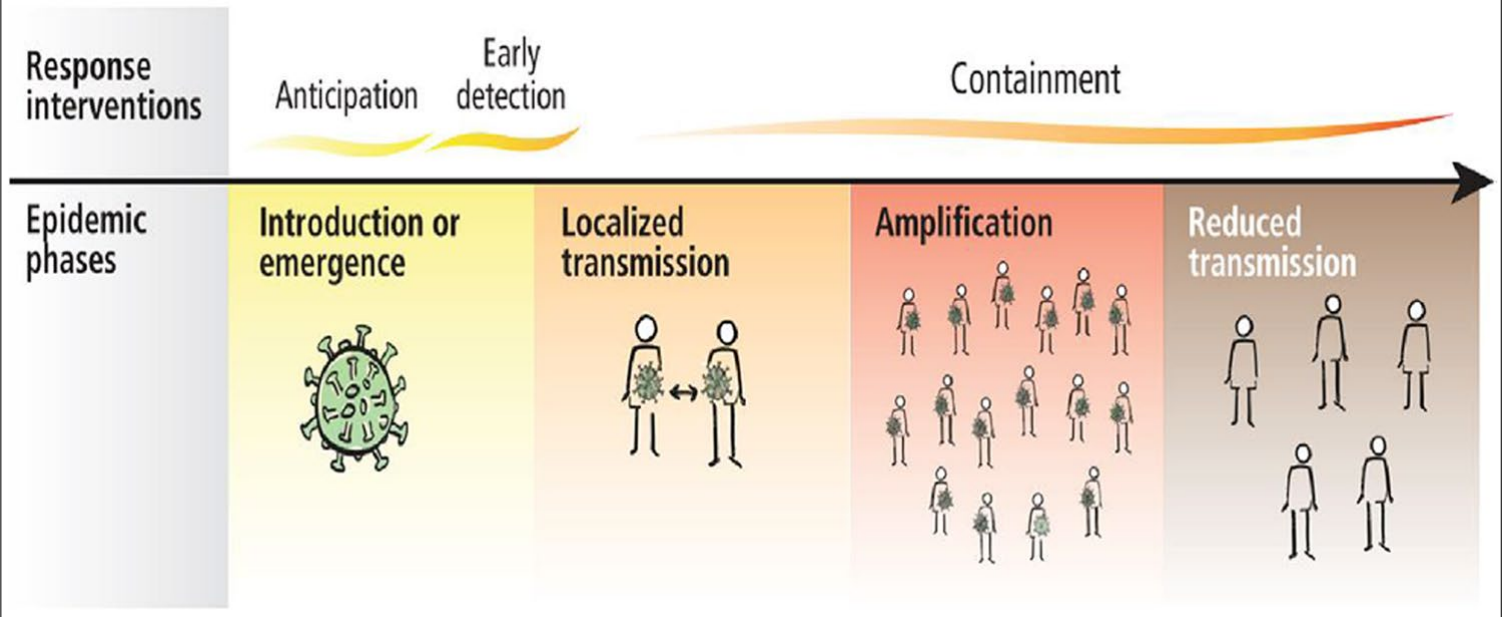
Spread of contiguous disease and with solution [5]

As social distancing reduces the rate of COVID-19 transmission to a great extent [9, 10], social distancing is one of the measures to slow the transmission of pandemic influenza shown in Fig. 1.

COVID-19 pandemic was reported on 30 January 2020 in India [1]. Transmission escalated during March, after occurrences of several cases in the country [11]. The initially reported cases had a background of travel history to affected countries. On 17 March 2020, the Indian Government advisory urges states to take social distancing [12].

The need for social distancing is because Covid-19 pandemic is due to the transmission of coronavirus from one infected person to another uninfected person [13, 14], either by direct contact, through droplets, or indirect contact to the contaminated surface [15]. COVID-19 has four transmission stages. In the first stage, it appears in travels from other countries. The second stage is local transmission. In the third stage, it is spread in the community which cannot be traced. And the fourth stage becomes an epidemic in the country [16].

The mathematical model develops by Signer Laboratory, a stem cell research laboratory located in Moores Cancer Center at the University of California San Diego has proven that if social distancing is neglected, then the effective reproduction number would be equal to the basic reproduction number [17].

According to the European Center for Disease Prevention and Control, the transmission rate in India is 1.81 [1, 3] on 31.03.2020 [12, 18]. In general, when the reproduction number is less than one that indicates not every infected person spreads the virus to another, the disease ceases to become an epidemic [19].

The below table validates the benefit of social distancing. The data are analyzed for three different scenarios. The scenarios are like no social distancing, a 50% reduction in social exposer, and a 75% reduction in social exposure. The researched data in Table 1 have shown that if the social distancing is increased up to 50%, then for 5 days infection rate reduces by 50%, and for a 30-day period the infection rate decreased significantly to 96%.

**Table 1:** Transmission rate of COVID-19 [17]

Scenario	5-day period	30-day period
No social distancing	1 infected person 2.5 * other	406 people infected
50% reduction in social exposure	1 infected person 1.25 * other	15 people infected
75% reduction in social exposure	1 infected person 0.625 * other	2.5 people infected

*For estimations only. It is not possible to infect only a fraction of another person

The methodology proposed in this paper to remind 1.5-m distancing from another person is by the use of PIR sensors and proposed wrist band.

The IoT (Internet of things) is an emerging platform that enables the communication between electronic devices and sensors using the Internet to enhance the living style [20]. Motion sensing technologies are used to detect movement in the vicinity. This is commonly used for security [21], industrial, and transportation systems [22, 23].

PIR sensor works by detecting the presence of thermal energy in confined spaces [24]. It calculates infrared light radiating from an object in its field of view. This is made up of a pyroelectric sensor that can detect different levels of radiation. When a temperature difference is detected by one of its beams, the sensor will be activated. When all the beam detects the same temperature again, the sensor will be deactivated. PIR sensors are similar to cameras which only can see warm things.

**Fig. 2:**
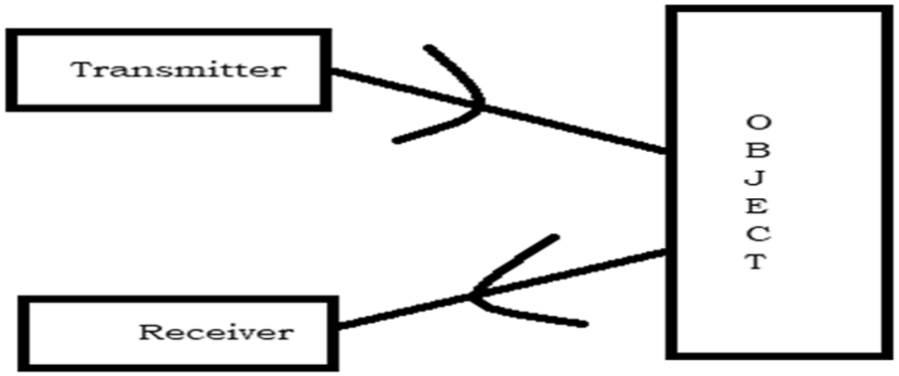
PIR sensor

The working principle of the PIR Sensor is shown in Fig. 2.

## Motivation

Lockdown is not a permanent solution to deal with COVID-19. Lockdown has affected the economy [25, 26] [12, 27, 28], daily routine as well as the mental health of the people [29]. After a certain stage, the government has to provide some relief in the lockdown. It will be a very cumbersome task to lift the lockdown at the end when all persons infected with COVID-19 get cured. Though such a scenario will be better, it is not feasible. So ultimately to adopt the regular practice of maintaining the distance of 1.5 m and hand sanitization is the better preventive against COVID-19 [13, 30].

In the unlocking process, WHO issued a guideline to maintain social distancing. This precaution is to be taken up by each individual while executing routine work. Due to reason, the need for a personal social distancing device is required. This leads to introducing either different techniques to maintain social distancing or different devices to maintain social distancing.

In some countries, the wristband with a Bluetooth device maintains the proximity measure. In some countries Wi-Fi, Bluetooth technology is used to implement social distancing. In India, social distancing is analyzed by using DRISHTI in the metros at public places [8].

In this, the social distance monitoring mechanism is controlled by the real-time camera mounted at a mall or the train station. This camera will capture real-time images of the crowd in the mall, town, and at New York Central Station. This uses the Oxford Town Center dataset, Mall dataset and Train Station dataset, and deep learning approach to detect the distance among persons in a crowd. This analyzes the social distancing in a crowd. This monitoring is called visual social distancing. The proposed system uses the fixed monocular camera so that distance among the persons can be traced using the region of interest. This uses the parameters as the camera to height, width, area of the region of interest on the ground plane, minimum physical distance also sending off the audiovisual cue, and threshold to check social distancing. YOLOv3 and Deepsort were proposed to detect and track the violation index for the non-social distance behavior for pedestrians. The distance estimation is done by the Euclidean distance between pose vectors [31].

This device is used to check the temperature of the human being. This used temperature sensor is an infrared temperature sensor. It uses ESP8266 Wifi Module to send the temperature of the human being to the system. The MQTT protocol is used to transfer the data to the server. Another hardware used in the indoor safety monitoring system is the mask detection system. This system is designed using Raspberry Pi and a camera module. This will also check whether the social distancing is maintained or not. This uses OpenCV for mask detection as well as a social distance monitoring system. This proposed system is designed based on an approach to monitor temperature as well as mask position, and if both are in an appropriate state, then the only user to come inside the building else mark the details of users and send the message will alert the violation of the rule [32].

From the work of the authors [31, 32], social distancing is monitored using cameras. These cameras check social distancing. This also checks the mask-wearing positions. The system alerts the persons who violate the social distancing. In [31], author suggested approach uses the deep learning concepts to monitor the position among the persons using YOLOv3 and Deepsort along with the audio signals, whereas in [32] it uses OpenCV to monitor the social distancing parameter.

This paper uses the wearable PIR sensor which will detect the human body temperature if the temperature is between 27 and 36 degree Celsius. If the temperature is more than 36 degree Celsius, it will send the alert message on mobile to the person who wore the wearable PIR device. This does not provide the solution to the social distancing management [33].

The use of cameras at public places such as a mall, train station, or in town finds the social distancing among the people. Using camera based social distancing alerting system people will notified, but this system is utilized at only at public places where the system is installed. This system does not assure the social distancing criteria among the individual other than the public places where this system is not in implementation. PIR sensor is economical to use, and this is used to detect the temperature of the human being. This could be used to detect the human presence in a vicinity. Hence the spread of the virus will be minimized and this will enhance the safety measure by maintaining the social distance criteria among the people in the surrounding.

Therefore to maintain social distancing measures in the public or private sector by considering the safety issue of an individual. In this suggested paper, a new approach was introduced, due to which each individual can use this device in the vicinity and will maintain the social distancing.

## Method

In the proposed technique, preventive measures to break the chain of transmission in the vicinity are a prime consideration. In the methodology of maintaining social distancing, the wristband with PIR sensors is proposed. The wristband helps in maintaining 1.5-m distancing and warns of sanitization in alternate cases.

**Fig. 3:**
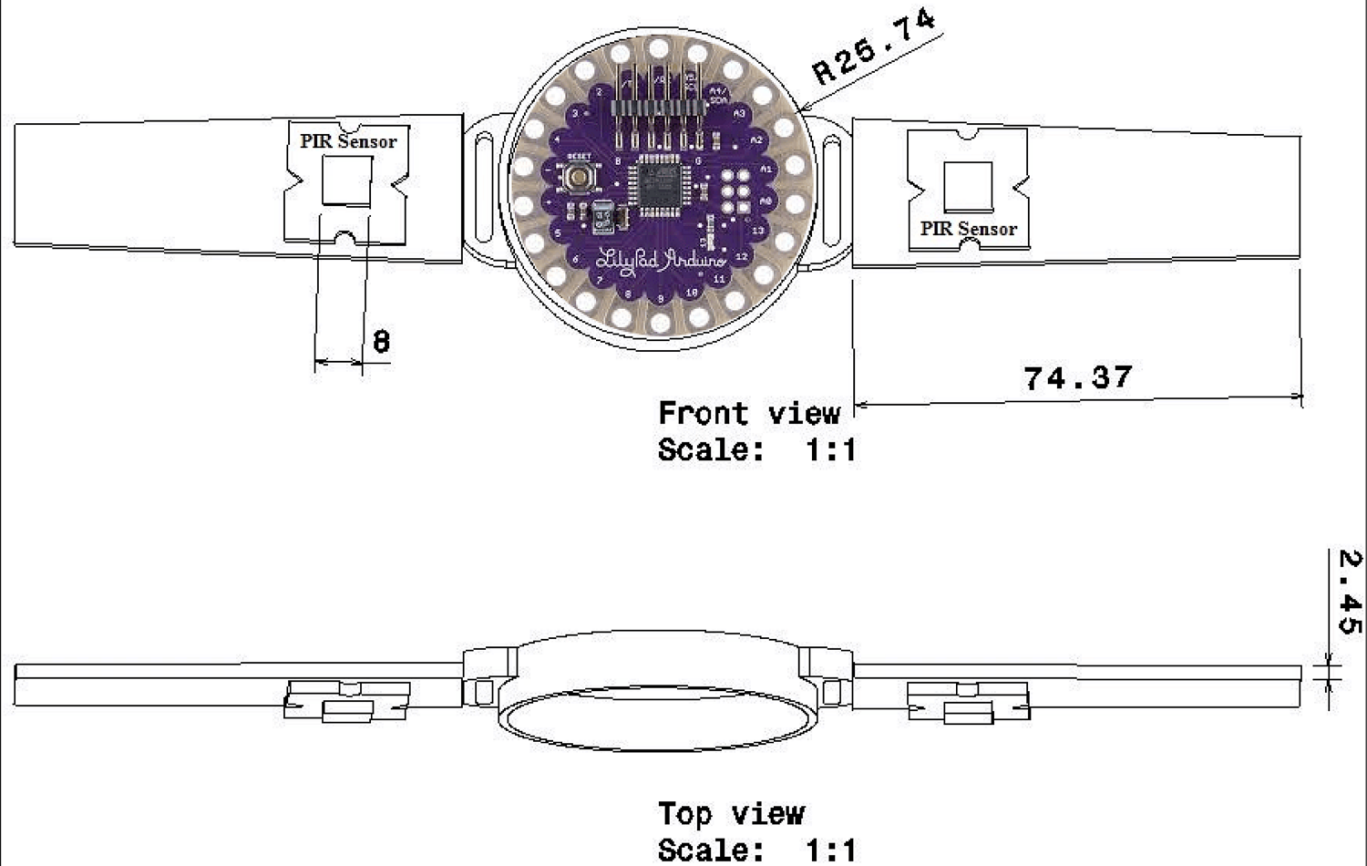
Schematic diagram of wristband with PIR sensor

**Fig. 4:**
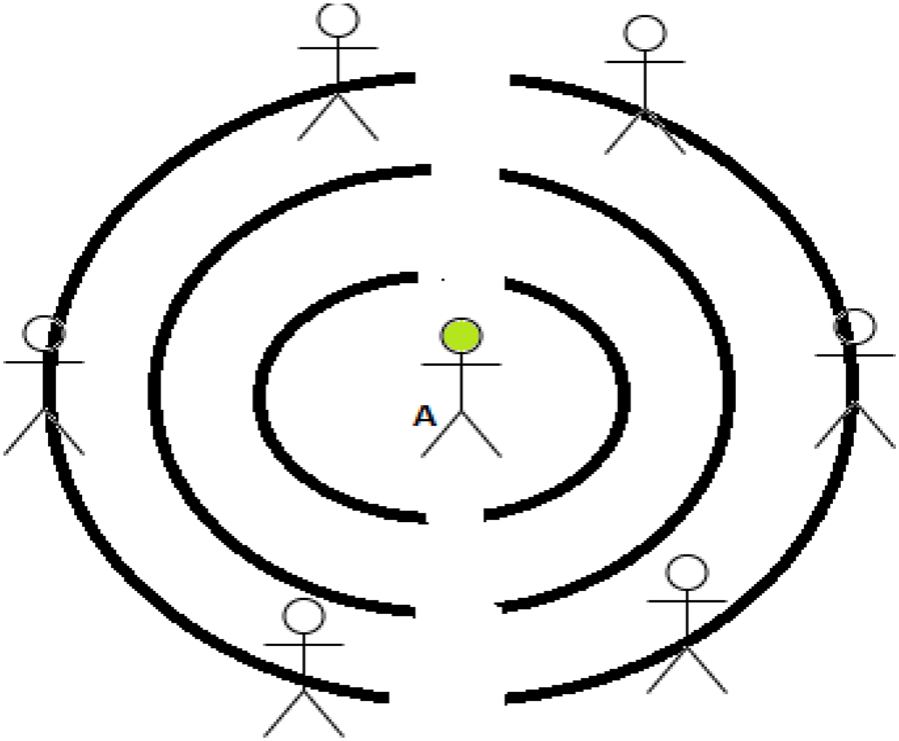
Working principle of wristband with PIR sensor

A wristband having PIR sensors will be developed for sensing humans in the vicinity of 1.5 m. The proposed PIR sensors fitted band is easily wearable on the wrist. The schematic design of the PIR sensor wristband and its working principle are shown in Figs. 3 and 4, respectively.

The wristband consists of Arduino (LilyPad), two PIR sensors, a speaker jack, buzzer, push button, and connecting wires. This wristband has a size of approximately 100 g including wristband belt and all electronic devices. The Arduino will act as a controlling unit and will control different operations such as sensing input signals, and according to the input, signal outputs will be generated as per programming. PIR sensors allow the sense of movement of an object in a range, and this detects different levels of infrared radiations. Speaker jack is provided to connect external speakers for audio output.

The speaker will help to convey an alert message to the user. The buzzer is used as an audio signaling device that alerts about people's presence in the vicinity within a certain distance. This buzzer will indicate the person who wears a wristband as well as the person who is breaking the social distancing guideline. The push button is provided to stop the buzzer as and when required. The stop button pressing ensures that the person is acknowledging and following the guideline issued by WHO for social distancing and hand sanitization. All the components are connected properly through wires.

A PIR sensor installed at the wristband will detect moving objects [21]. This will detect the moving object. After detecting an object in a range of 2 m, it will send the audio message as “person is present at a distance of 2 m.” When that person comes closer at a distance of 1 m, then it will send an altering signal that the person needs to sanitize. The detailed working of the proposed wristband is elaborated on below.

The proposed wristband will detect moving objects [34] in all directions within the vicinity of 1.5 m. In this, the PIR sensor will detect the human presence after sensing through the front as well as the back sensor. This will send the sensor signal to the Arduino. Arduino will continuously take input through these PIR sensors and be processed as per the threshold value for the distancing as 2 m and 1.5 m. If the distance sensed will be 2 m, then it will issue an audio signal which will provide an alert about the distance. And if the person comes in a range of 1.5 m or less, then it will send an audio signal and send the signal to the buzzer. This buzzer will indicate both people who have wristbands and who have not to wear the wristband. Also, the buzzer will be continuing to ring until it will be stopped by the stop button. Because of this, the persons in the vicinity will try to maintain the distancing criteria of at least 1.5 m. Also, the audio message will be issued to maintain distance and sanitize hand. The stop button will not be enabled till 60 s after the 1.5-m buzzer is pressed. To stop the ringing of the buzzer, one will need to press a stop button. A time limit of 60 s is set up for the buzzer alerting, considering that within this period the person will sanitize his or her hands for at least 20 s. The buzzer can be stopped manually after the sanitization process.

This system will always scan the presence of a person in a range of 1.5 m. If the person presents in a range of 1.5 m, then it will send an audio signal. Due to this in the same instance, both persons will get an alert. This alert will be given to the person not only who is carrying the device but also a person who will not have the device. This will reduce the rate of transmission which is caused because of either negligence or unawareness of social distancing as per guidelines suggested by WHO.

The reason behind the selection of two criteria as 2 m and 1.5 m is [3, 14]:

1. If the person is not wearing a mask, then droplets after sneezing and coughing will travel a distance up to 2 m, so to reduce this possibility of transmission.
2. If the person is wearing a mask, still some droplets may travel up to 1.5 m after sneezing and coughing. So sanitization will reduce any possibility of being infected.

Consider that person A has worn a wristband containing two PIR sensors. The PIR sensor will sense the human presence by considering the thermal energy of the object. When any person will be detected by these PIR signals in the range of 2 or 1.5-m, audio signals will be generated. When person A will be in a safe zone, it will not generate any message.

## Proposed algorithm

The proposed algorithm will effectively provide a solution for physical distancing using the PIR sensor. The pseudo-code of the proposed algorithm is mentioned as follows:

### Algorithm:

To detect human/object in the range of physical distancing.

1. Input: Human /object in a range of PIR sensors.
2. Output: Alerting an audio signal to the user.
3. Senses the presence of humans using the PIR sensor module.
4. If a human/moving object is detected, then check for the distance.
5. If the distance is less than 2 m, then alert the audio message for the distance only.
6. If the distance is equal to 1.5 m, then alert message and warn for safe distancing and sanitization.
7. The message will be continued till the person is not sanitizing. After the sanitization, person has to press the reset button.

**Fig. 5:**
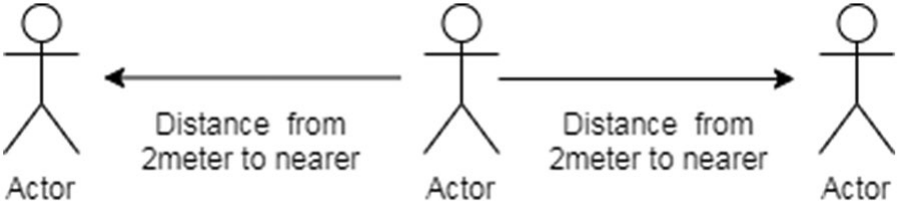
Social distancing among individuals 5

PIR motion sensor is a low-cost device used to detect a change in the vicinity within or between 15 and 20 feet. The output of the IR sensor is an analog signal; signal changes are dependent on the distance between the sensor and an object (Fig. 5).

The Grove PIR motion sensor measures the distance with response speed 0.3 s to 25 s. This will measure a distance maximum of 3–6 m. This sensor will detect the object with an angle of 120 degrees.

## Discussion

Widely seen social distancing technique on public places is either by marking by color or manual distancing by assuming 1.5-m distancing.

Also, Aarogya Setu App has been launched as an initiative by the Government of India, to provide medical assistance to the people. This assistance is in the form of asking for symptoms to the user for COVID-19. If the symptoms match COVID -19, then it suggests a medical test. If the user results from COVID-19 positive, then it stores information of that particular person into the database. Also, the Aarogya Setu app determines the risk of contacting a COVID-19-infected person within six feet of distance by scanning in the database of known cases across India. Aarogya Setu app needs either GPS or Bluetooth and a smartphone with the Aarogya Setu app.

Aarogya Setu app needs data from the user, and if the data are not made available, then it will not provide any assistance. On the other hand, a country like India has lots of issues like awareness and availability of resources (in the case of smartphones).

Every time it is not possible for school students, children, old people, disabled people, or working people in the areas which have banned the use of a smartphone or for the people in the weaker section to carry a smartphone. This wristband will ensure the safety of these people if they use it. The band also suggests safety instructions for the people who have not worn this band but will come in the vicinity of a person having the band.

The transmission rate of infection in COVID-19 is calculated by using the following formula:

$$Infected\;People = (Transmissionrate) \wedge Days$$

By using this device on day one, we can decrement in the transmission rate. The device is used by the single infected person, and when it will be in the vicinity of the other person, the buzzer will start. Similarly, for the person who is not infected wearing the wrist band the probability of being infected also minimizes.

## Result

**Fig. 6:**
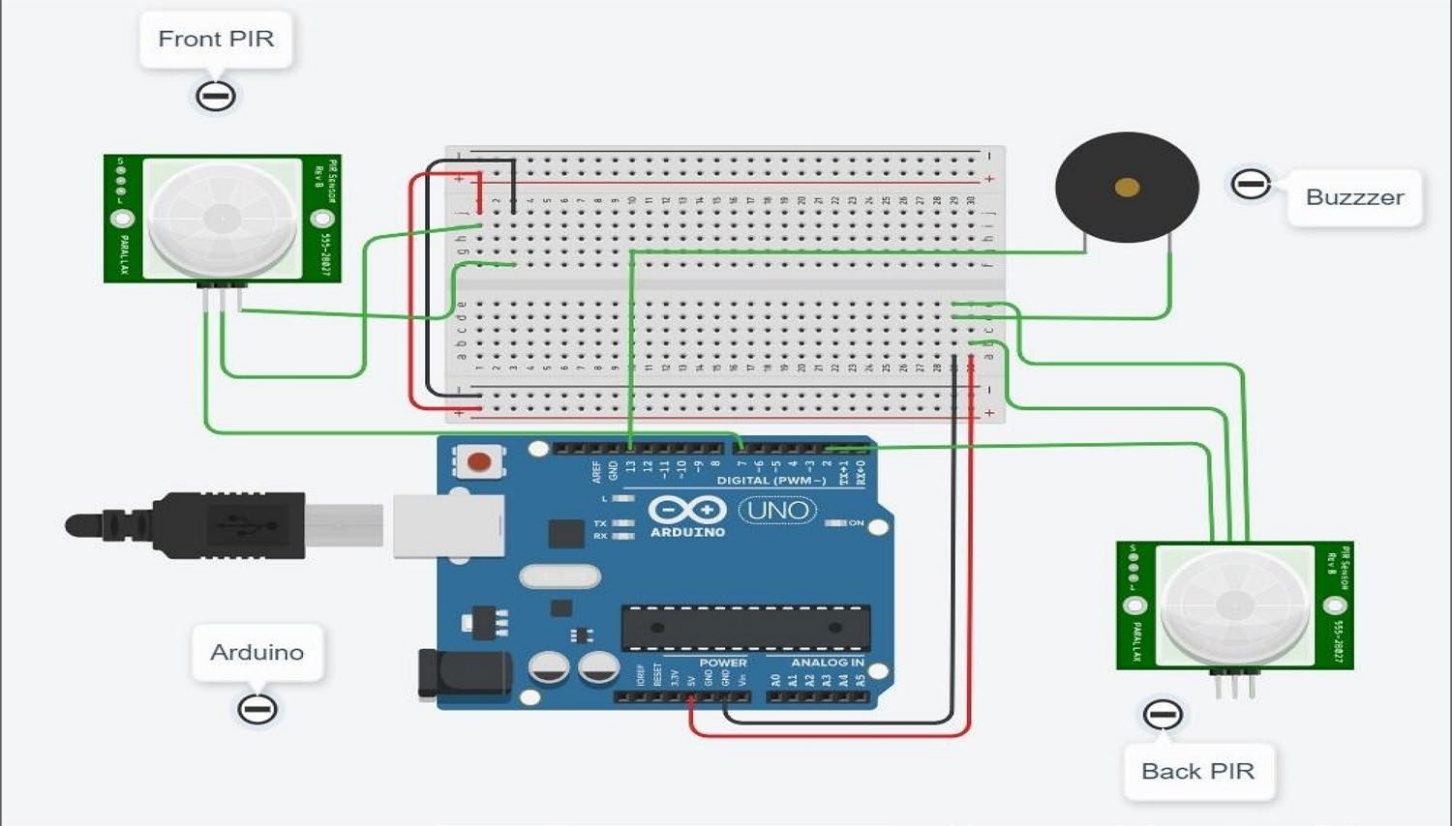
Arduino Uno with front and back PIR sensor with buzzer

**Fig. 7:**
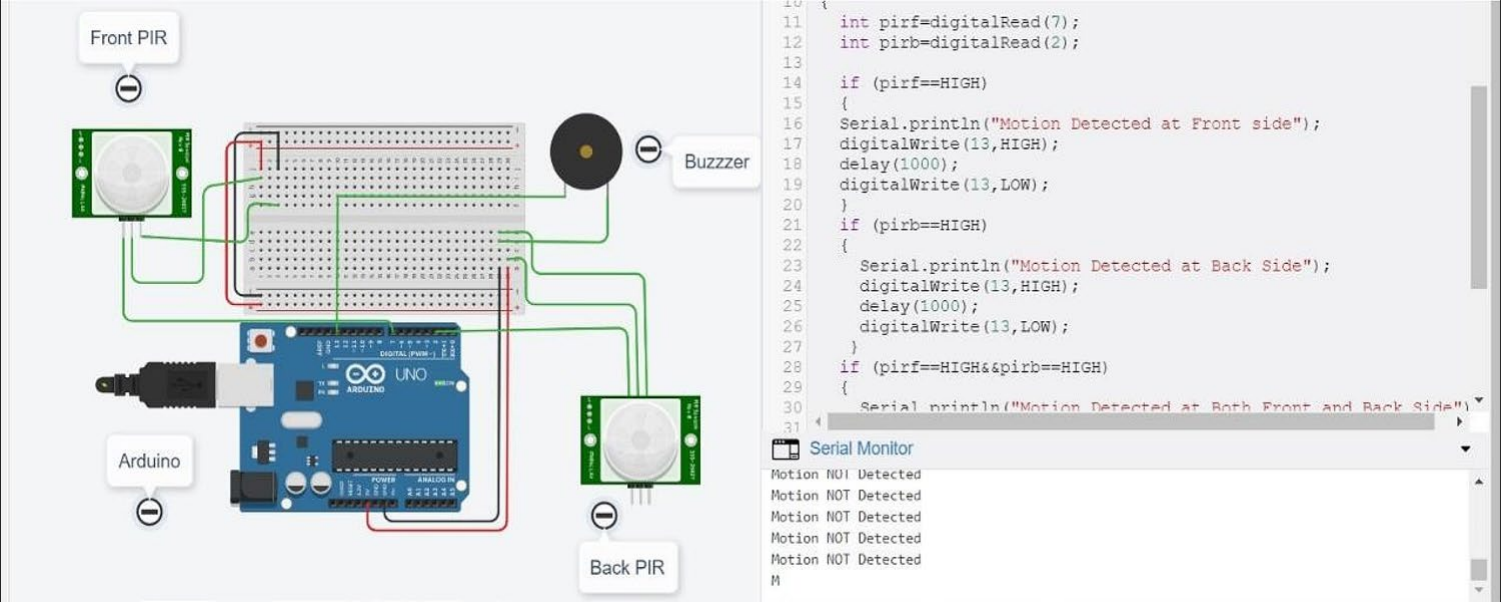
Output at an instance when no object detected

**Fig. 8:**
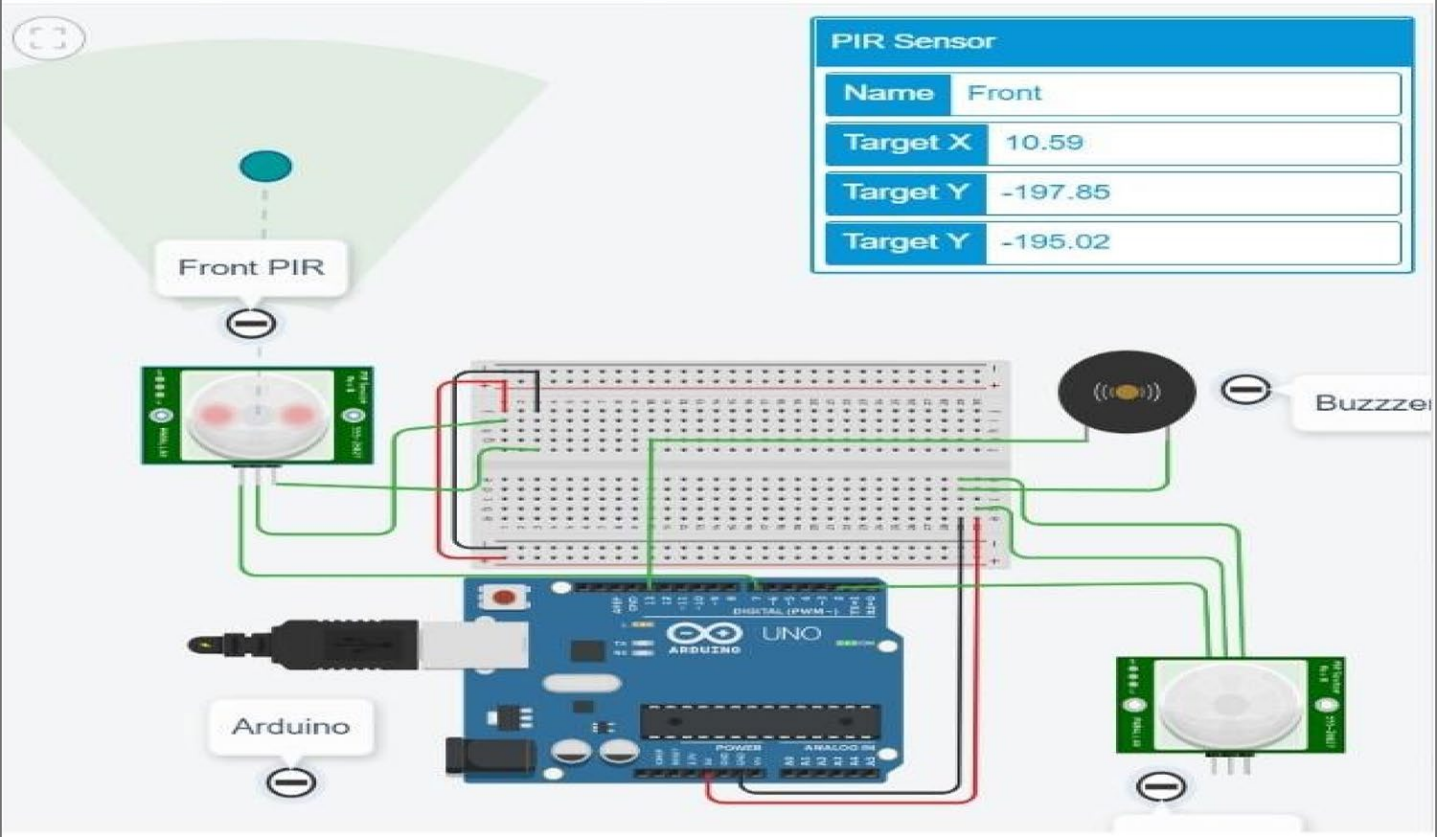
Output at an instance when object detected at front PIR

**Fig. 9:**
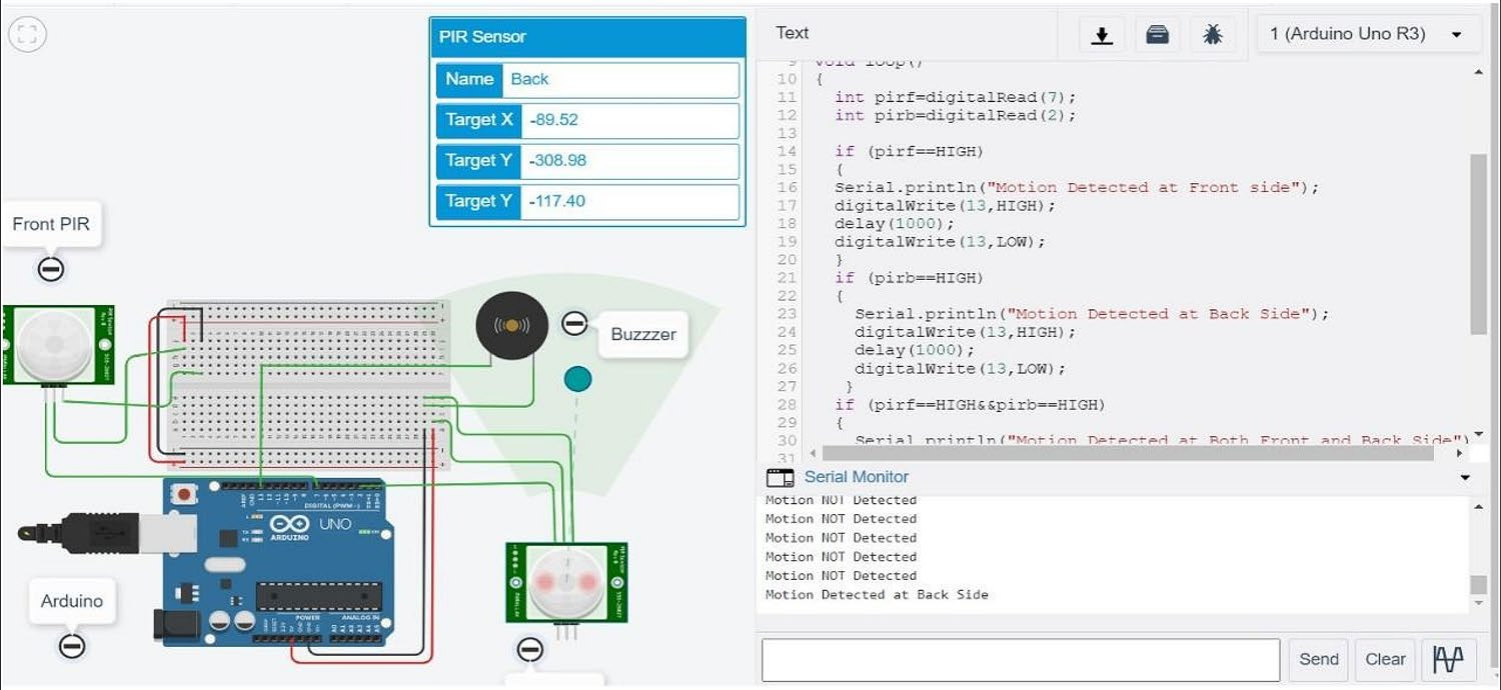
Output at an instance when object detected at back PIR

The results are simulated on Tinkercad Simulator. For simulation purpose, results are tested on Arduino Uno which is a universal Arduino. The circuit diagram is shown in Fig. 6. Figure 7 shows the circuit and code with no object detected at front PIR and Back PIR sensors. The introduced delay for both sensors is 1000 ms. Figure 8 shows that the object is human at the front PIR. Similar results are observed in the back PIR sensors in Fig. 9.

The same code and same devices are compatible with the Arduino LilyPad [35]. This Arduino LilyPad is mainly designed for wearable products only. Hence, this device will be worn by the user at his/her wrist. This has an in-build rechargeable battery. Also, the used PIR sensor is the Grove Mini PIR [36] sensor to detect human presence in a vicinity. This wristband can be easily worn by men or women in a vicinity**.**

## Conclusion

Lockdown cannot be implemented permanently, and this is not the ultimate solution to stop the spread of the COVID-19 virus. Despite lockdown, we need to concentrate on such a machine due to which we can minimize the spread of the virus. Hence, this suggested device will be a preventive measure to avoid the spread of the virus by wearing every individual in a vicinity. This device can be worn by any individual. And after resuming the work in the unlocking phase, we need to maintain social distancing in the proximity along with mask and sanitization practice. But maximum people are not sufficiently assured about maintaining social distancing. To this problem, the solution is provided through the PIR wrist. This wristband will continuously monitor and alert to reduce the possibility of being infected. This will be a very useful and better solution to minimize the possibility of being infected because of ignorance of social distancing.

## Data Availability

No data and material are used in experiments.

## Abbreviations

COVID-19: Coronavirus disease 2019
SARS-CoV-2: Severe acute respiratory syndrome coronavirus 2
WHO: World Health Organization
PIR: Passive Infrared
